# Cross-border mobility in the Meuse-Rhine Euroregion: impact of COVID-19 border restrictions on everyday activities and visiting social network members

**DOI:** 10.3389/fpubh.2024.1281072

**Published:** 2024-04-18

**Authors:** Céline J. A. van Bilsen, Stephanie Brinkhues, Christian J. P. A. Hoebe, Christina Stabourlos, Chrissy P. B. Moonen, Stefaan Demarest, Daniëlle A. T. Hanssen, Inge H. M. van Loo, Paul H. M. Savelkoul, Dirk Philippsen, Brigitte A. M. van der Zanden, Nicole H. T. M. Dukers-Muijrers

**Affiliations:** ^1^Department of Social Medicine, Maastricht University, Care and Public Health Research Institute (CAPHRI), Maastricht, Netherlands; ^2^Department of Sexual Health, Infectious Diseases, and Environmental Health, Living Lab Public Health, Public Health Service South Limburg, Heerlen, Netherlands; ^3^Department of Knowledge & Innovation, Public Health Service South Limburg, Heerlen, Netherlands; ^4^Department of Medical Microbiology, Infectious Diseases & Infection Prevention, Maastricht University Medical Center (MUMC+), Maastricht, Netherlands; ^5^Department of Epidemiology and Public Health, Sciensano, Brussels, Belgium; ^6^Care and Primary Health Research Institute (CAPHRI), Maastricht University, Maastricht, Netherlands; ^7^Gesundheitsberichterstattung, Gesundheitsamt Düren, Düren, Germany; ^8^Foundation euPrevent, Heerlen, Netherlands; ^9^Department of Health Promotion, Care and Public Health Research Institute (CAPHRI), Maastricht University, Maastricht, Netherlands

**Keywords:** cross-border mobility, border regions, COVID-19 pandemic, travel restrictions, surveys and questionnaires, logistic models, social health, social networks

## Abstract

**Introduction:**

Cross-border mobility (CBM) to visit social network members or for everyday activities is an important part of daily life for citizens in border regions, including the Meuse-Rhine Euroregion (EMR: neighboring regions from the Netherlands, Belgium, and Germany). We assessed changes in CBM during the COVID-19 pandemic and how participants experienced border restrictions.

**Methods:**

Impact of COVID-19 on the EMR’ is a longitudinal study using comparative cross-border data collection. In 2021, a random sample of the EMR-population was invited for participation in online surveys to assess current and pre-pandemic CBM. Changes in CBM, experience of border restrictions, and associated factors were analyzed using multinomial and multivariable logistic regression analysis.

**Results:**

Pre-pandemic, 82% of all 3,543 participants reported any CBM: 31% for social contacts and 79% for everyday activities. Among these, 26% decreased social CBM and 35% decreased CBM for everyday activities by autumn 2021. Negative experience of border restrictions was reported by 45% of participants with pre-pandemic CBM, and was higher (p < 0.05) in Dutch participants (compared to Belgian; aOR= 1.4), cross-border [work] commuters (aOR= 2.2), participants with cross-border social networks of friends, family or acquaintances (aOR= 1.3), and those finding the measures ‘limit group size’ (aOR= 1.5) and ‘minimalize travel’ (aOR= 2.0) difficult to adhere to and finding ‘minimalize travel’ (aOR= 1.6) useless.

**Discussion:**

CBM for social contacts and everyday activities was substantial in EMR-citizens, but decreased during the pandemic. Border restrictions were valued as negative by a considerable portion of EMR-citizens, especially when having family or friends across the border. When designing future pandemic control strategies, policy makers should account for the negative impact of CBM restrictions on their citizens.

## Introduction

The European Union (EU) comprises 360 internal land border regions, encompassing 40% of the territory and 38% of the EU population (1, 2). Until March 2020, there was complete border permeability among the signatories of the Schengen Agreement (3). The coronavirus disease 2019 (COVID-19) pandemic, which started in 2020, presented an unprecedented situation. The temporary reintroduction of border control resulted in a sudden restriction of free movement in the EU and had far-reaching health, social, and economic consequences (3). This situation also affected the border region Meuse-Rhine Euroregion (EMR), covering the border area of the Netherlands, Belgium, and Germany.

Mobility in the EMR is synonymous with cross-border mobility (CBM) due to the population living in close proximity to a national border. Mobility is crucial for accessing facilities and engaging in social interactions, and thereby various aspects of wellbeing and health. For example, in the EMR, CBM encompasses essential medical activities such as visiting healthcare professionals. Within the EMR there is a high degree of patient mobility and citizens tend to be highly willing to travel to neighboring member states to receive medical treatments (4). Border control measures during the pandemic hindered cross-border healthcare, resulting in challenges in cross-border communication and collaboration among EMR public health professionals (5).

Furthermore, CBM has various transport modes. Those that require physical activity, such as walking and cycling, have been shown to positively impact self-perceived health, mental health including perceived stress, loneliness, social health, and reduced mortality (6, 7). Additionally, car use and use of public transportation have been associated with health outcomes such as increased physical activity, reduced social frailty in elderly, and reduced loneliness in European adults (6, 8, 9). Limited mobility during the COVID-19 pandemic has been linked to reduced happiness, also referred to as subjective wellbeing (10, 11). Finally, mobility is associated with increased social participation (12). Maintaining CBM is thus important in health.

With a population of 4 million, the inhabitants of the EMR have extensive cross-border connections, including social contacts, work, study, leisure, and healthcare (3, 5, 13–16). Therefore, CBM is an important part of daily life for EMR-citizens and borders are generally perceived as non-existent (17). Citizens cross borders for several reasons which are interconnected, such as cross-border commuting, social visits, and leisure activities (18). This includes everyday activities such as grocery shopping and visiting restaurants, and in-person contact with social network members with visits to family, friends, and acquaintances living across the border (social visits). Social connections plays a crucial role in promoting physical and mental well-being, including happiness (19, 20). Social isolation is associated with health risks, chronic illness, and all-cause mortality (21, 22). Social networks can serve as a buffer by providing social support during stressful events, including the COVID-19 pandemic (23). However, social interactions have been shown to be significantly decreased during the pandemic, with fewer in-person contacts and reduced social network interactions (23).

The efficacy of border restrictions in controlling the transmission of SARS-CoV-2 has been debated. The effectiveness of border restrictions depends highly on timing, the stage of the epidemic, interconnectedness of countries, local measures undertaken, extent of implementation, and adherence (24, 25). A study among 10,0001 citizens in the Dutch EMR subregion indicated that CBM was not associated with seroprevalence of SARS-CoV-2, which may suggest a limited role of border traffic in the virus spread (16).

By our knowledge, there are no existing reports on CBM for visiting social network members and everyday activities in the EMR. Whilst it is known that CBM decreased during the pandemic (15), it is not clear to which extent, and how citizens experienced CBM restrictions.

This study aimed to assess changes in cross-border visits during the pandemic and the impact of border restrictions on citizens in the EMR, focusing on differences between the three regions. Therefore, we assessed factors associated with changes in CBM for social contacts and everyday activities in autumn of 2021, compared to before the COVID-19 pandemic, and which factors are associated with experienced negative impact of border restrictions.

## Methods

### Context of border restrictions in the EMR during the COVID-19 pandemic

**Figure 1:**
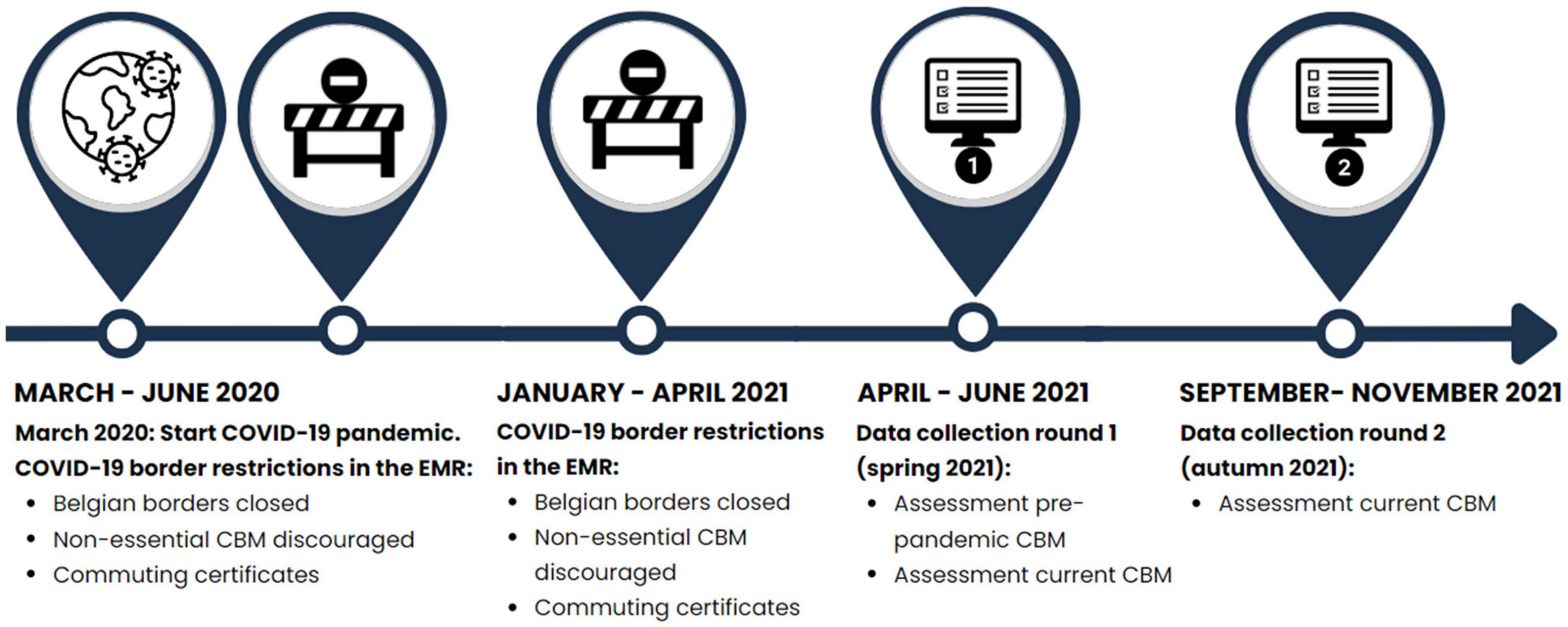
Timeline of data collection and border restrictions in the EMR.

Belgium closed its borders with neighboring countries between March 2020 and June 2020, and restricted all entry to the country. Border checks were enforced and border crossings had to be justified via forms. Cross-border workers were allowed to cross borders, but only with a declaration from the employer, which posed challenges for independent workers. Germany never introduced border controls with Belgium or the Netherlands, but health checks were enforced from March 2020. The Netherlands never closed its borders (15). However, all three EMR-countries advised citizens to stay within their own country (26). Between January and April 2021 Belgium again closed its borders for non-essential travel (16) (Figure 1).

### Study design and participants

**Figure 2:**
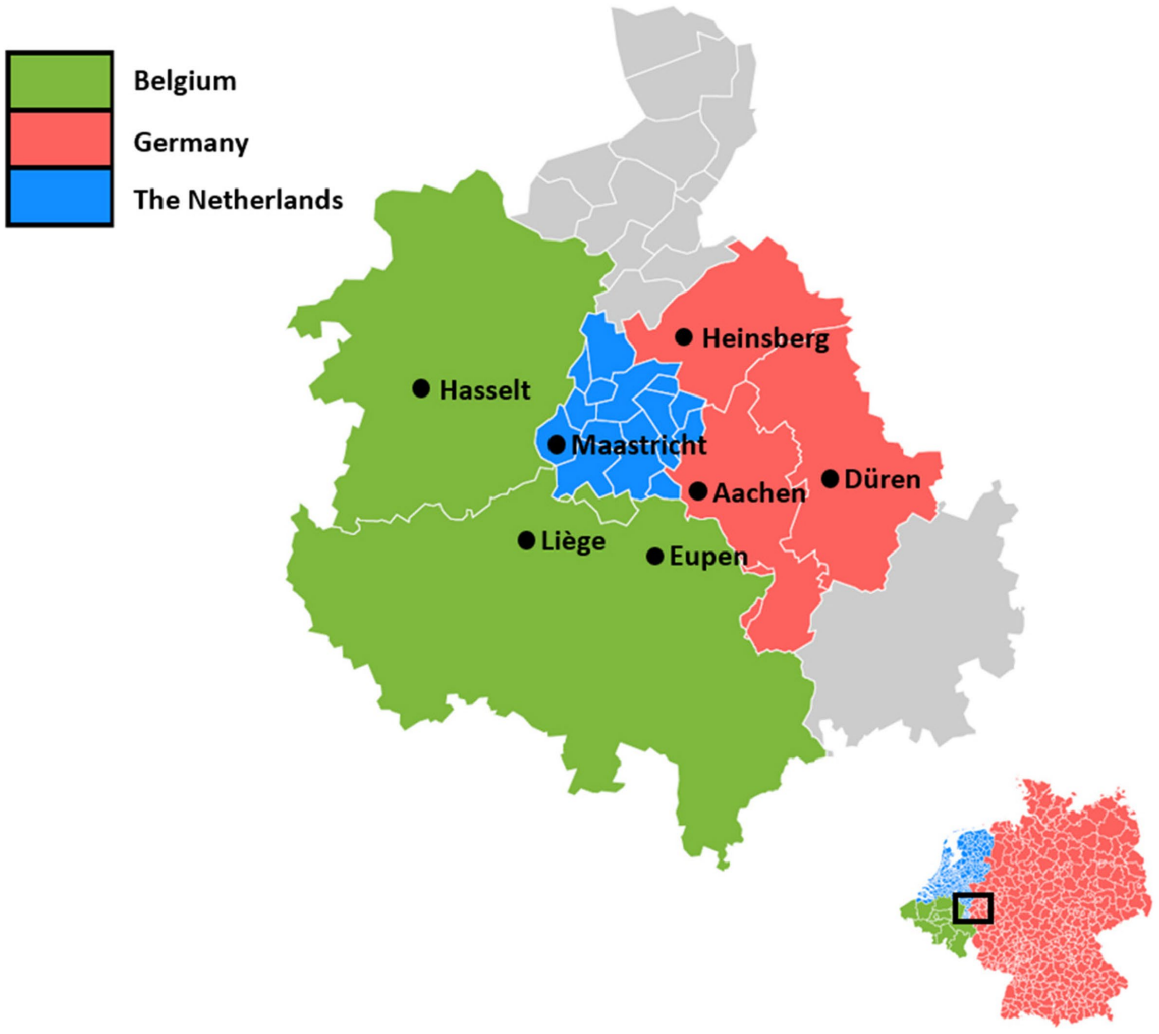
The parts of the Meuse-Rhine Euroregion (EMR) included in this study.

‘Impact of COVID-19 on the EMR’ is a longitudinal study of EMR residents, using a comparative cross-border data collection (27). A survey was conducted in EMR-subregions, including the Belgian provinces Limburg (Dutch-speaking), Liège (French-speaking), and the German-speaking community of Belgium (Ostbelgien as a part of Liège). Apart from that, South Limburg (the Netherlands; Dutch-speaking) and the German City of Aachen and Districts of Düren and Heinsberg (German-speaking) were included (Figure 2).

Participants were recruited through written invitations, including a letter and questionnaire link, based on national resident registers. In spring 2021, a random sample of in total 26,925 citizens aged 18 years and older, residing in private households in the EMR, was selected from the national registers in each country and invited to participate. Further details about the sampling methods applied in each country, stepwise selection, and opt-out procedures has been described previously (27). Participation rates ranged from 15.3% in the Belgian subregions (8,911 invitees and 1,366 participants) to 27% (11,266 invitees and 3,042 participants) and 26.7% (6,748 invitees and 1,598 participants) in the Dutch and German subregions, respectively. All 6,006 initial participants (round one) were subsequently invited for follow-up (round two).

Questionnaire data on CBM were collected between April 13 and June 29, 2021 (round one) and between September 21 and November 20, 2021 (round two; Figure 1).

A total of 3,557 participants completed the questionnaires from both rounds. In the current study, CBM behavior and impact of border restrictions are described for the 3,543 participants who had no missing data for variables included in the analysis.

### Measurements

#### CBM pre-pandemic (outcome 1)

Participants reported whether they have family, friends or acquaintances in another EMR-country and how many times per month on average they crossed the border to either visit social network members or for everyday activities before the pandemic. Based on the distribution of the data, the number of total cross-border visits before the pandemic was grouped into 0 times per month, 1–2 times per month, 3–5 times per month, and ≥ 6 times per month.

We distinguished between different types of CBM:

- *Cross-border visits for visiting social network members*: this was the average (as reported by the participant) number of times a month participants visited family, friends or acquaintances living across the border before the pandemic (measured in round one) and in the last month (round two).
- *Cross-border visits for everyday activities*: this was the average (as reported by the participant) number of times a month a participant had crossed the border for a short visit (for example grocery shopping or visiting a restaurant), before the COVID-19 pandemic, i.e., before February 2020 (measured in round one) and in the last month (when filling in the round 2 questionnaire in September to November 2021).
- *Total visits* were assessed by combining information on cross-border visits for social contacts or everyday activities.

Work-related CBM was not included as an outcome since changes herein were not evaluated and the number of participants working across the border was low.

#### Changes in CBM during the pandemic (outcome 2)

For CBM for social contacts (outcome 2a), everyday activities (outcome 2b), and total visits (outcome 2), participants were categorized into three groups based on their reported number of cross-border visits per month in autumn 2021 compared to before the COVID-19 pandemic: those with a decrease of more than one, those with an increase of more than one, and those with no change (a change of one or zero).

#### Experience of border restrictions (outcome 3)

Experienced impact of border restrictions was assessed by a question on whether the experience of border restrictions during the pandemic was negative for the participant themselves (five-point scale; from totally disagree to totally agree). For analyses, this outcome was grouped into ‘neutral/no negative experience’ (answers: neutral, disagree or totally disagree) and ‘negative experience’ (answers: totally agree or agree).

#### CBM and happiness (sub analysis)

Given that mobility and wellbeing are closely linked, we conducted a sub analysis to explore potential associations between happiness and cross-border visits. Data on happiness (‘All things considered, how happy would you say you are?’), measured on a scale from 0 (unhappy) to 10 (happy), were collected for the pre-pandemic time point (assessed retrospectively in spring 2021) and in autumn 2021. A score < 8 was classified as indicative of unhappiness.

#### Covariates

Various socio-demographic factors were included: country (the Netherlands, Belgium or Germany), sex (male or female), age group (18–29, 30–39, 40–49, 50–59, 60–69 or ≥ 70 years), and level of education (theoretical or practical). Employment status was categorized into working in own country, working in another country, and not working. Apart from that, presence of comorbidities (yes/no) and having family, friends or acquaintances across the border (yes/no) were assessed.

Several measures that were taken in response to the COVID-19 pandemic were included in the questionnaire, such as ‘limit group size’, ‘minimalize travel’, and ‘work from home’. We assessed perceived usefulness and perceived difficulty of these measures as measured in round one (at time when the measures were implemented). For perceived usefulness of measures, options were ‘(very) useful or neutral’ and ‘not useful (at all)’. Perceived difficulty was divided into ‘(very) easy or neutral’ and ‘(very) difficult’.

### Analytical approach and methodology

Data on CBM were measured for three different countries with seven different regions. In analyses, the focus was on countries (not regions) due to the low number of participants in some regions. In all analyses, we evaluated CBM for social contacts, everyday activities, and total visits.

#### CBM pre-pandemic (outcome 1)

CBM for total visits, visiting social network members, and for everyday activities was described. Subsequently, we described the characteristics of participants who had CBM for social visits or everyday activities compared to those who did not have CBM before the pandemic. Characteristics of participants with or without CBM before the pandemic were described in a table of N and proportions. Univariate and multivariable logistic regression analyses were used to determine whether differences between country of residence and other covariates were statistically significant.

Subsequently, we restricted further analyses to those participants who reported CBM pre-pandemic.

#### Changes in CBM during the pandemic (outcome 2)

Changes in total CBM, social CBM (outcome 2a), and CBM for everyday activities (outcome 2b) were examined using descriptive analyses.

To determine factors associated with changes in total CBM since baseline (decrease, increase or no change [+/− 1]), univariate and multivariable multinomial regression analyses were used. The main determinant was country. Covariates evaluated were the number of cross-border visits pre-pandemic, sex, age group, level of education, work situation, presence of comorbidities, having family, friends or acquaintances across the border, and perceived usefulness and perceived efficacy for the measures ‘limit group size’, ‘minimalize travel’, and ‘work from home’.

Changes in CBM and associated factors was assessed for social visits (outcome 2a) and visits for everyday activities (outcome 2b) separately, restricted to the subpopulations who those who reported pre-pandemic social CBM or CBM for everyday activities. For these two outcomes, the number of pre-pandemic cross-border visits was included as a continuous variable, since participant numbers were smaller.

#### Experience of border restrictions (outcome 3)

To examine experience of border restrictions, univariate and multivariable logistic regression analyses were performed. Main determinants were country and changes in CBM (also defined as outcome 2). Other factors included were the number cross-border visits pre-pandemic, sex, age group, level of education, work situation, presence of comorbidities, having family, friends or acquaintances across the border, and perceived usefulness and perceived efficacy for the measures ‘limit group size’, ‘minimalize travel’, and ‘work from home’.

#### CBM and happiness (sub analysis)

We used descriptive statistics, chi-square tests, and logistic regression analysis to assess the proportions of participants classified as unhappy, comparing different groups based on the average number of cross-border visits per month (0 [reference], 1 to 2, 3 to 5, or > 5). We performed univariate analysis, followed by adjusted analysis taking into account country, age, sex, and having social contacts across the border.

### Statistical procedures and model building

For all main outcomes, univariate and multivariable models were adjusted for country and number of pre-pandemic cross-border visits. Apart from that, we corrected for changes in CBM for outcome 3. There was no multicollinearity between the included variables since Variance Inflation Factor (VIF) values were < 3. For pre-pandemic CBM, interactions between country and sex, as well as country and age, were tested and showed no statistically significant interaction. For model building, variables with a *p*-value of <0.10 in the univariate analysis were included in the multivariable models by backwards selection. A p-value of <0.05 was considered statistically significant to be retained in the final models. Data were analyzed with IBM Statistical Package for the Social Sciences (SPSS) version 27.

## Results

### Study population

A total of 3,543 participants completed both questionnaires and had no missing data for the variables included in the analysis. Participants were on average 55 ± 15.5 years old and 59% was female. The majority of included participants was Dutch (*N* = 1791; 51%), followed by German (*N* = 1,030; 29%), and Belgian (*N* = 722; 20%).

### Pre-pandemic CBM (outcome 1)

Of all 3,543 participants, 82% reported pre-pandemic CBM: 31% had at least one visit per month to cross-border social network members and 79% for everyday activities (Table 1). Differences in pre-pandemic CBM between the three countries were statistically significant in adjusted models (Supplementary Table 1). CBM was highest in the Netherlands (90% of participants; aOR = 4.4; 95%CI: 3.5–5.5), followed by Germany (81% of participants; aOR = 2.4; 95%CI 1.9–3.0), and Belgium (63% of participants; reference). CBM was >75% for all age groups. Participants who were employed more often reported CBM, with the highest proportion (96%) among participants working in another country. Most participants with pre-pandemic CBM had 1–2 cross-border visits per month (Table 1).

**Table 1:** Proportions for pre-pandemic CBM in participants living in the EMR (*N* = 3,543).

	CBM at baseline *N* = 2,900	%	No CBM at baseline *N* = 643	%	Total
Country					
Belgium	457	63.3	265	36.7	722
Netherlands	1,610	89.9	181	10.1	1791
Germany	833	80.9	197	19.1	1,030
Sex					
Female	1,698	82.0	373	18.0	2071
Male	1,202	81.7	270	18.3	1,472
Age group					
18–29	244	77.2	72	22.8	316
30–39	296	81.5	67	18.5	363
40–49	420	84.3	78	15.7	498
50–59	710	83.1	144	16.9	854
60–69	788	83.6	155	16.4	943
≥70	442	77.7	127	22.3	569
Level of education					
Theoretical	1,377	82.5	292	17.5	1,669
Practical	1,523	81.3	351	18.7	1874
Work situation					
Working in own country	1,595	84.2	299	15.8	1894
Working in other country	68	95.8	3	4.2	71
Not working	1,237	78.4	341	21.6	1,578
Presence of comorbidities					
No comorbidities	1,160	82.2	252	17.8	1,412
Comorbidities	1740	81.7	391	18.3	2,131
Having family/friends/acquaintances across the border					
Yes	1,277	94.0	82	6.0	1,359
No	1,623	74.3	561	25.7	2,184
Mobility at baseline (before the pandemic)					
Yes, social visits and visits for everyday activities	1,001	100	0	0	1,001
Yes, only visits for everyday activities	1806	100	0	0	1806
Yes, only social visits	93	100	0	0	93
No	0	0	643	100	643
Mobility in round 1 (spring 2021)					
Yes, social visits and visits for everyday activities	218	98.6	3	1.4	221
Yes, only visits for everyday activities	717	95.7	32	4.3	749
Yes, only social visits	130	98.5	2	1.5	132
No	1835	75.2	606	24.8	2,441
Mobility in round 2 (autumn 2021)					
Yes, social visits and visits for everyday activities	525	98.5	8	1.5	533
Yes, only visits for everyday activities	1,522	89.9	171	10.1	1,693
Yes, only social visits	108	90.8	11	9.2	119
No	745	62.2	453	37.8	1,198
Number of pre-pandemic border crossings per month					
0	0	0	643	100	643
1–2	1,318	100	0	0	1,318
3–5	882	100	0	0	882
≥6	700	100	0	0	700

### Changes in CBM (outcome 2)

**Figure 3:**
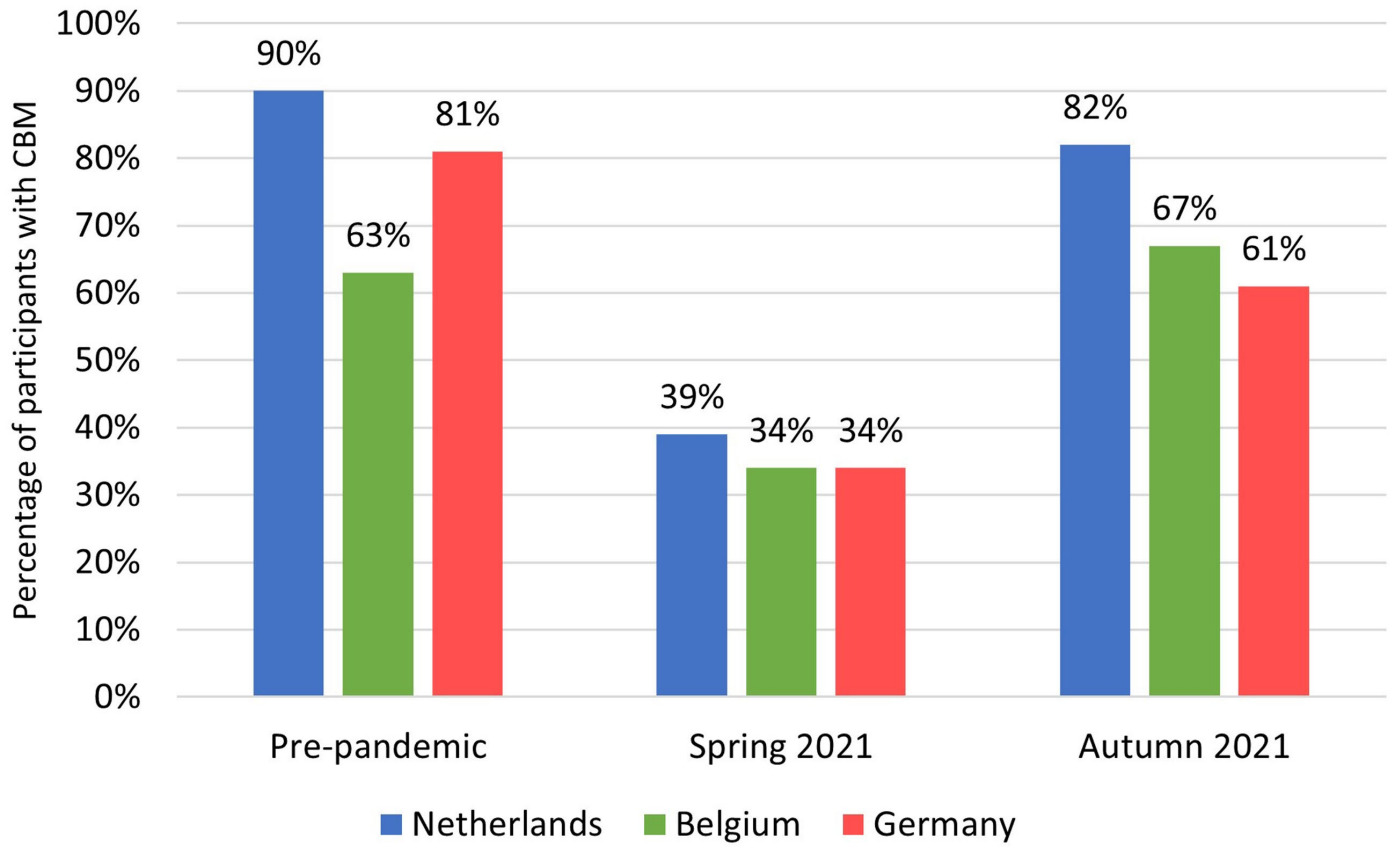
Proportion of CBM per EMR-country before the pandemic and in spring and autumn 2021 for participants with pre-pandemic CBM (*N* = 2900).

Pre-pandemic CBM was reported by 2,900 participants (82%). Of those, 37% reported CBM in spring 2021 (round 1) and 74% reported CBM in autumn 2022 (round 2; Figure 3; Supplementary Table 2).

A decrease in cross-border visits (assessed at round 2) was observed in 40% of participants with pre-pandemic CBM, whereas 49% had no change or a change of one, and 11% had an increase of more than one (Table 2). The majority of participants (>90%) had a change between 2 and 5.

**Table 2:** Proportions and multinomial regression analysis for changes in cross-border visits in participants with pre-pandemic CBM (*N* = 2,900).

	Proportions				Univariate-corrected for country and number of pre-pandemic cross-border visits per month						Multivariable					
	Decrease > 1 N = 1,146		Increase > 1 N = 324		Decrease > 1			Increase > 1			Decrease > 1			Increase > 1		
	N	%	N	%	OR	95%CI	*p*	OR	95%CI	*p*	OR	95%CI	*p*	OR	95%CI	*p*
**Country**							**0.001**			**0.001**			**<0.001**			**<0.001**
Belgium	146	31.9	37	8.1	Ref			Ref			Ref			Ref		
Netherlands	680	42.2	209	13.0	0.92	0.69–1.23	0.58	1.62	1.10–2.39	**0.014**	0.89	0.67–1.18	0.41	1.79	1.20–2.66	**0.004**
Germany	320	38.4	78	9.4	1.20	0.88–1.63	0.24	1.25	0.82–1.92	0.30	1.15	0.85–1.57	0.37	1.35	0.87–2.08	0.18
**Number of pre-pandemic border crossings per month**							**<0.001**			**<0.001**			**<0.001**			**<0.001**
1–2	160	12.1	140	10.6	Ref			Ref			Ref			Ref		
3–5	445	50.5	107	12.1	8.83	7.06–11.04	**<0.001**	2.20	1.66–2.93	**<0.001**	9.04	7.22–11.32	**<0.001**	2.12	1.59–2.83	**<0.001**
≥6	541	77.3	77	11.0	44.08	32.93–59.01	**<0.001**	6.26	4.36–9.00	**<0.001**	47.28	35.02–63.82	**<0.001**	5.31	3.65–7.73	**<0.001**
**Sex**							**<0.001**			**<0.001**			**<0.001**			**<0.001**
Female	677	39.9	158	9.3	Ref			Ref			Ref			Ref		
Male	469	39.0	166	13.8	0.78	0.64–0.94	**0.011**	1.40	1.09–1.80	**0.008**	0.78	0.64–0.95	**0.014**	1.39	1.08–1.79	**0.010**
**Age group**							0.41			0.41						
18–29	78	32.0	33	13.5	0.92	0.61–1.39	0.68	1.66	0.98–2.81	0.059						
30–39	108	36.5	31	10.5	1.14	0.78–1.68	0.50	1.37	0.81–2.32	0.25						
40–49	166	39.5	37	8.8	1.10	0.77–1.56	0.61	1.11	0.67–1.84	0.68						
50–59	278	39.2	85	12.0	0.99	0.72–1.34	0.92	1.45	0.95–2.22	0.089						
60–69	325	41.2	99	12.6	1.12	0.83–1.52	0.46	1.57	1.04–2.39	**0.033**						
≥70	191	43.2	39	8.8	Ref			Ref								
**Level of education**							0.11			0.11						
Theoretical	518	37.6	159	11.5	Ref			Ref								
Practical	628	41.2	165	10.8	1.21	1.00–1.47	**0.049**	1.01	0.78–1.29	0.97						
**Work situation**							**<0.001**			**<0.001**			**<0.001**			**<0.001**
Working in own country	606	38.0	189	11.8	Ref			Ref			Ref			Ref		
Working in other country	35	51.5	18	26.5	0.75	0.36–1.55	0.43	2.90	1.35–6.23	**0.007**	0.74	0.36–1.55	0.43	2.84	1.32–6.13	**0.008**
Not working	505	40.8	117	9.5	0.94	0.78–1.15	0.56	0.74	0.57–0.97	**0.026**	0.94	0.77–1.14	0.54	0.76	0.58–0.98	**0.035**
**Presence of comorbidities**							0.25			0.25						
No comorbidities	440	37.9	142	12.2	Ref			Ref								
Comorbidities	706	40.6	182	10.5	1.09	0.90–1.32	0.40	0.87	0.68–1.12	0.29						
**Having family/friends across the border**							0.31			0.31						
No	506	31.2	166	10.2	Ref			Ref								
Yes	640	50.1	158	12.4	1.00	0.82–1.23	1.00	1.22	0.93–1.58	0.15						
**Perceived difficulty (R1)**																
**Limit group size**							**0.033**			**0.033**						
(very) easy/neutral	945	40.1	249	10.6	Ref			Ref								
(very) difficult	201	36.9	75	13.8	0.79	0.62–1.01	0.063	1.17	0.87–1.58	0.30						
**Minimalize travel**							0.11			0.11						
(very) easy/neutral	968	39.0	265	10.7	Ref			Ref								
(very) difficult	178	42.7	59	14.1	0.98	0.75–1.29	0.90	1.39	0.99–1.94	0.055						
**Work from home** ^**A**^							**0.023**			**0.023**						
(very) easy/neutral/ not applicable	843	40.0	215	10.2	Ref			Ref								
(very) difficult	303	38.2	109	13.7	1.04	0.84–1.28	0.75	1.44	1.11–1.88	**0.007**						
**Perceived usefulness (R1)**																
**Limit group size**							**<0.001**			**<0.001**			**0.003**			**0.003**
(very) useful/neutral	1,049	40.1	272	10.4	Ref			Ref			Ref			Ref		
(very) useless	97	33.8	52	18.1	0.74	0.54–1.03	0.076	1.61	1.13–2.30	**0.009**	0.76	0.55–1.06	0.11	1.51	1.05–2.16	**0.027**
**Minimalize travel**							**0.002**			**0.002**						
(very) useful/neutral	1,074	39.8	287	10.6	Ref			Ref								
(very) useless	72	35.3	37	18.1	0.72	0.49–1.06	0.098	1.63	1.07–2.48	**0.022**						
**Work from home** ^**A**^							**0.014**			**0.014**						
(very) useful/neutral/not applicable	1,082	39.8	292	10.7	Ref			Ref								
(very) useless	64	35.4	32	17.7	0.76	0.51–1.14	0.19	1.54	0.99–2.40	0.056						

Reference = no change in short cross-border visits. OR, odds ratio; 95%CI = 95% confidence interval. Nagelkerke pseudo-R^2^ value of the multivariable model = 0.38. Decrease > 1 indicates a decrease of 1 or more cross-border visits per month in autumn 2021, compared to before the COVID-19 pandemic. Increase > 1 indicates an increase of 1 or more cross-border visits per month in autumn 2021, compared to before the COVID-19 pandemic. R1 = round 1 questionnaire. ^A^For ‘work from home’, all participants who were not working (e.g., retired, students) were grouped ‘not applicable’. This group was then included in the categories of ‘(very) useful or neutral’ for perceived usefulness, and ‘(very) easy or neutral’ for perceived efficacy of working from home.

#### Factors associated with changes in CBM (outcome 2)

Around 40% of participants from the Netherlands and Germany experienced a decrease in CBM, compared to 32% in Belgium. Dutch participants most often had an increase in CBM (13%), followed by Belgian (8%) and German (9%) participants (Table 2).

Factors independently associated with a decrease of >1 in cross-border visits in autumn 2021, compared to baseline were having 3–5 (aOR = 9.0; 95%CI 7.2–11.3) or ≥ 6 (aOR = 47.3; 95%CI 35.0–63.8) baseline border crossings per month and female sex.

Factors independently associated with an increase of >1 in cross-border visits were Dutch residency (aOR = 1.8; 95%CI 1.2–2.7) as opposed to Belgian, male sex (aOR = 1.4; 95%CI 1.1–1.8), working across the border (aOR = 2.8; 95%CI 1.3–6.1), and low perceived usefulness of the measure ‘limit group size’ (aOR = 1.5; 95%CI 1.1–2.2). A higher number of pre-pandemic cross-border visits was also a significant factor for an increase in CBM (3–5 visits: aOR = 2.1; 95%CI 1.6–2.8; ≥6 visits: aOR = 5.3; 95%CI = 3.7–7.7; Table 2).

#### Factors associated with changes in CBM to visit social network members (outcome 2a)

Participants in the Netherlands and Germany presented similar proportions of around 25% for a decrease in social CBM in autumn 2021, compared to pre-pandemic. Those with a decrease of >1 in social CBM had an average number of pre-pandemic border crossings of 12.46, whereas it was 10.09 for those with an increase. This was the only factor associated with a decrease in social CBM between autumn 2021 and baseline (aOR = 1.2; 95%CI 1.1–1.2). Factors independently associated with an increase of >1 in cross-border visits were working across the border (aOR = 4.1; 95%CI 1.6–10.4), low perceived usefulness of ‘minimalize travel’ (aOR = 2.1; 95%CI 1.1–3.8) and a higher number of pre-pandemic border crossings per month (aOR = 1.1; 95%CI 1.1–1.2; Table 3).

**Table 3:** Proportions and multinomial regression analysis for changes in cross-border visits to visit social network members in participants with pre-pandemic CBM for visiting social network members (*N* = 1,094).

	Proportions				Univariate-corrected for country and number of pre-pandemic long cross-border visits per month						Multivariable					
	Decrease > 1 N = 287		Increase > 1 N = 68		Decrease > 1			Increase > 1			Decrease > 1			Increase > 1		
	N	%	N	%	OR	95%CI	*p*	OR	95%CI	*p*	OR	95%CI	*p*	OR	95%CI	*p*
**Country**							0.54			0.54			0.95			0.95
Belgium	32	25.0	12	9.4	Ref			Ref			Ref			Ref		
Netherlands	187	26.7	40	5.7	1.35	0.81–2.24	0.25	0.70	0.35–1.43	0.33	1.18	0.70–2.00	0.53	1.08	0.48–2.45	0.85
Germany	68	25.7	16	6.0	1.35	0.77–2.37	0.30	0.78	0.34–1.75	0.54	1.19	0.67–2.12	0.56	1.27	0.51–3.18	0.61
**Number of pre-pandemic border crossings per month (continuous)**	12.46 (10.51)		10.09 (9.94)				**<0.001**			**<0.001**			**<0.001**			**<0.001**
					1.17	1.14–1.21	**<0.001**	1.14	1.10–1.19	**<0.001**	1.18	1.14–1.21	**<0.001**	1.12	1.08–1.17	**<0.001**
**Sex**							0.92			0.92						
Female	130	27.7	31	6.6	Ref			Ref								
Male	157	25.1	37	5.9	0.94	0.69–1.28	0.69	0.99	0.60–1.66	0.98						
**Age group**							0.31			0.31						
18–29	14	21.5	4	6.2	0.76	0.36–1.62	0.48	1.15	0.33–4.05	0.83						
30–39	15	15.0	10	10.0	0.56	0.28–1.12	**0.099**	2.03	0.77–5.32	0.15						
40–49	46	28.7	10	6.3	1.05	0.62–1.77	0.85	1.29	0.50–3.34	0.60						
50–59	75	28.1	12	4.5	0.96	0.60–1.53	0.86	0.87	0.35–2.16	0.76						
60–69	87	28.4	23	7.5	1.11	0.71–1.74	0.64	1.72	0.76–3.89	0.19						
≥70	50	25.5	9	4.6	Ref			Ref								
**Level of education**							0.43			0.43						
Theoretical	127	23.6	34	6.3	Ref			Ref								
Practical	160	28.8	34	6.1	1.22	0.90–1.66	0.20	1.05	0.63–1.75	0.85						
**Work situation (R1)**							**0.005**			**0.005**			**0.004**			**0.004**
Working in own country	134	24.2	29	5.2	Ref			Ref			Ref			Ref		
Working in other country	18	31.6	14	24.6	0.66	0.29–1.50	0.32	3.73	1.49–9.31	**0.005**	0.64	0.28–1.46	0.29	4.10	1.62–10.38	**0.003**
Not working	135	28.0	25	5.2	1.16	0.85–1.58	0.35	1.04	0.59–1.82	0.90	1.14	0.83–1.56	0.42	1.14	0.64–2.02	0.65
**Presence of comorbidities**							0.31			0.31						
No comorbidities	98	23.8	28	6.8	Ref			Ref								
Comorbidities	189	27.7	40	5.9	1.27	0.92–1.74	0.14	0.98	0.58–1.64	0.92						
**Perceived difficulty (R1)**
**Limit group size**							0.31			0.31						
(very) easy/neutral	241	27.1	55	6.2	Ref			Ref								
(very) difficult	46	22.5	13	6.4	0.73	0.49–1.10	0.13	0.93	0.49–1.79	0.83						
**Minimalize travel**							**0.027**			**0.027**			**0.021**			**0.021**
(very) easy/neutral	242	26.5	48	5.3	Ref			Ref			Ref			Ref		
(very) difficult	45	25.0	20	11.1	0.80	0.53–1.22	0.31	1.94	1.08–3.45	**0.025**	0.81	0.53–1.25	0.35	2.07	1.14–3.75	**0.016**
**Work from home** ^**A**^							0.91			0.91						
(very) easy/neutral/not applicable	207	25.6	50	6.2	Ref			Ref								
(very) difficult	80	28.2	18	6.3	0.94	0.66–1.32	0.71	0.91	0.51–1.62	0.75						
**Perceived usefulness (R1)**
**Limit group size**							0.45			0.45						
(very) useful/neutral	263	26.5	59	5.9	Ref			Ref								
(very) useless	24	23.5	9	8.8	0.84	0.49–1.43	0.52	1.45	0.67–3.11	0.35						
**Minimalize travel**							0.16			0.16						
(very) useful/neutral	263	25.8	58	5.7	Ref			Ref								
(very) useless	24	31.6	10	13.2	1.09	0.61–1.97	0.77	2.21	1.02–4.78	**0.045**						
**Work from home** ^**A**^							0.16			0.16						
(very) useful/neutral/ not applicable	269	26.5	59	5.8	Ref			Ref								
(very) useless	18	22.8	9	11.4	0.73	0.39–1.37	0.32	1.74	0.79–3.83	0.17						

Reference = no change in long cross-border visits for visiting social network members. OR, odds ratio; 95%CI = 95% confidence interval. Nagelkerke pseudo-R^2^ value of the multivariable model = 0.24. Decrease > 1 indicates a decrease of 1 or more long cross-border visits to visit family, friends or acquaintances per month in autumn 2021, compared to before the COVID-19 pandemic. Increase > 1 indicates an increase of 1 or more long cross-border visits to visit family, friends or acquaintances per month in autumn 2021, compared to before the COVID-19 pandemic. R1 = round 1 questionnaire. ^A^For ‘work from home’, all participants who were not working (e.g., retired, students) were grouped ‘not applicable’. This group was then included in the categories of ‘(very) useful or neutral’ for perceived usefulness, and ‘(very) easy or neutral’ for perceived efficacy of working from home.

#### Factors associated with changes in CBM for everyday activities (outcome 2b)

In autumn 2021, around 35% of Dutch and German participants and 28% of Belgian participants showed a decrease in CBM for everyday activities, compared to pre-pandemic. Dutch participants most often had an increase in CBM (11%; Table 4).

**Table 4:** Proportions and multinomial regression analysis for changes in short cross-border visits for everyday activities in participants with pre-pandemic CBM for everyday activities (*N* = 2,789).

	Proportions				Univariate-corrected for country and number of pre-pandemic cross-border visits for everyday activities per month						Multivariable					
	Decrease > 1 N = 967		Increase > 1 N = 261		Decrease > 1			Increase > 1			Decrease > 1			Increase > 1		
	N	%	N	%	OR	95%CI	*p*	OR	95%CI	*p*	OR	95%CI	*p*	OR	95%CI	*p*
**Country**							**0.008**			**0.008**			**0.010**			**0.010**
Belgium	122	27.7	30	6.8	Ref			Ref			Ref			Ref		
Netherlands	568	36.7	176	11.4	1.19	0.91–1.57	0.20	1.56	1.02–2.37	**0.039**	1.14	0.87–1.50	0.36	1.74	1.12–2.71	**0.014**
Germany	277	34.6	55	6.9	1.38	1.03–1.84	**0.033**	1.07	0.67–1.73	0.78	1.26	0.94–1.69	0.12	1.17	0.71–1.92	0.53
**Number of pre-pandemic border crossings per month (continuous)** Mean (SD)	7.86 (6.97)		5.75 (6.96)				**<0.001**			**<0.001**			**<0.001**			**<0.001**
					1.36	1.32–1.41	**<0.001**	1.30	1.25–1.35	**<0.001**	1.43	1.38–1.48	**<0.001**	1.30	1.25–1.36	**<0.001**
**Sex**							**<0.001**			**<0.001**			**<0.001**			**<0.001**
Female	559	34.6	119	7.4	Ref			Ref			Ref			Ref		
Male	408	34.8	142	12.1	0.91	0.76–1.10	0.32	1.57	1.20–2.06	**0.001**	0.91	0.75–1.09	0.30	1.57	1.20–2.07	**0.001**
**Age group**							0.77			0.77						
18–29	72	31.6	26	11.4	1.02	0.69–1.53	0.91	1.69	0.95–2.98	0.072						
30–39	91	31.8	20	7.0	0.99	0.68–1.42	0.94	0.96	0.53–1.75	0.90						
40–49	139	33.9	33	8.0	0.97	0.70–1.36	0.87	1.06	0.63–1.80	0.82						
50–59	239	34.4	70	10.1	0.99	0.74–1.33	0.96	1.35	0.86–2.11	0.20						
60–69	274	36.3	77	10.2	1.09	0.82–1.45	0.57	1.34	0.86–2.09	0.20						
≥70	152	36.6	35	8.4	Ref			Ref								
**Level of education**							0.98			0.98						
Theoretical	453	34.1	126	9.5	Ref			Ref								
Practical	514	35.2	135	9.3	1.00	0.84–1.20	0.98	0.97	0.74–1.28	0.84						
**Work situation**							**<0.001**			**<0.001**			**<0.001**			**<0.001**
Working in own country	526	33.8	147	9.5	Ref			Ref			Ref			Ref		
Working in other country	29	45.3	14	21.9	0.20	0.09–0.47	**<0.001**	1.00	0.41–2.41	1.00	0.21	0.086–0.49	**<0.001**	1.01	0.41–2.44	0.99
Not working	412	35.2	100	8.5	0.93	0.78–1.12	0.46	0.82	0.62–1.09	0.18	0.94	0.78–1.13	0.49	0.83	0.63–1.10	0.20
**Presence of comorbidities**							0.89			0.89						
No comorbidities	382	34.1	109	9.7	Ref			Ref								
Comorbidities	585	35.1	152	9.1	1.00	0.83–1.20	1.00	0.94	0.71–1.23	0.65						
**Having family/friends across the border**							**<0.001**			**<0.001**			**<0.001**			**<0.001**
No	504	31.4	122	7.6	Ref			Ref			Ref			Ref		
Yes	463	39.1	139	11.7	0.57	0.46–0.69	**<0.001**	0.94	0.70–1.26	0.66	0.57	0.46–0.70	**<0.001**	0.94	0.70–1.26	0.68
**Perceived difficulty (R1)**
**Limit group size**							0.14			0.14						
(very) easy/neutral	785	34.7	198	8.7	Ref			Ref								
(very) difficult	182	34.7	63	12.0	1.06	0.84–1.34	0.61	1.39	1.01–1.92	**0.044**						
**Minimalize travel**							0.20			0.20						
(very) easy/neutral	814	34.1	211	8.8	Ref			Ref								
(very) difficult	153	38.0	50	12.4	0.98	0.75–1.27	0.85	1.35	0.95–1.93	0.099						
**Work from home** ^**A**^							0.073			0.073						
(very) easy/neutral/not applicable	700	34.8	174	8.6	Ref			Ref								
(very) difficult	267	34.5	87	11.2	1.03	0.84–1.26	0.76	1.40	1.05–1.87	**0.023**						
**Perceived usefulness (R1)**																
**Limit group size**							**0.009**			**0.009**			**0.032**			**0.032**
(very) useful/neutral	880	35.0	219	8.7	Ref			Ref			Ref			Ref		
(very) useless	87	31.3	42	15.1	0.92	0.68–1.26	0.60	1.73	1.18–2.55	**0.005**	0.93	0.68–1.27	0.65	1.61	1.09–2.37	**0.017**
**Minimalize travel**							**0.027**			**0.027**						
(very) useful/neutral	904	34.9	233	9.0	Ref			Ref								
(very) useless	63	31.7	28	14.1	0.73	0.50–1.07	0.11	1.41	0.89–2.24	0.14						
**Work from home** ^**A**^							0.10			0.10						
(very) useful/neutral/ not applicable	911	34.9	237	9.1	Ref			Ref								
(very) useless	56	32.0	24	13.7	0.76	0.51–1.13	0.18	1.32	0.81–2.15	0.27						

Reference = no change in short cross-border visits for everyday activities. OR, odds ratio; 95%CI = 95% confidence interval. Nagelkerke pseudo-R^2^ value of the multivariable model = 0.29. Decrease > 1 indicates a decrease of 1 or more short cross-border visits for everyday activities (e.g., grocery shopping, visiting restaurants) per month in autumn 2021, compared to before the COVID-19 pandemic. Increase > 1 indicates an increase of 1 or more short cross-border visits for everyday activities (e.g., grocery shopping, visiting restaurants) per month in autumn 2021, compared to before the COVID-19 pandemic. R1, round 1 questionnaire. ^A^For ‘work from home’, all participants who were not working (e.g., retired, students) were grouped ‘not applicable’. This group was then included in the categories of ‘(very) useful or neutral’ for perceived usefulness, and ‘(very) easy or neutral’ for perceived efficacy of working from home.

Participants with cross-border social contacts more often had an increase or decrease in CBM for everyday activities. This factor was associated with lower likeliness for a decrease (aOR = 0.6; 95%CI 0.5–0.7). Furthermore, working across the border was associated with lower odds for a decrease (aOR = 0.2; 95%CI 0.09–0.5). A higher number of pre-pandemic border crossings was associated with higher odds for both a decrease (aOR = 1.4; 95%CI 1.4–1.5) and increase (aOR = 1.3; 95%CI 1.3–1.4) in CBM for everyday activities. Other factors independently associated with an increase were Dutch residency, compared to Belgian (aOR = 1.7; 95%CI 1.1–2.7), male sex (aOR = 1.6; 95%CI 1.2–2.1), and low perceived usefulness of ‘limit group size’ (aOR = 1.6; 95%CI 1.1–2.4; Table 4).

### Outcome 3: experienced negative impact of border restrictions

Out of the 2,900 participants with pre-pandemic CBM, 1299 (45%) reported having experienced the border restrictions during the pandemic as negatively for themselves (Table 5).

**Table 5:** Proportions and logistic regression analysis for negative experience with border restrictions in participants with baseline CBM (*N* = 2,900).

	Negative experience of border restrictions		Univariate-corrected for country, change in cross-border visits, and number of pre-pandemic cross-border visits per month			Multivariable		
	N = 1,299	%	OR	95%CI	*p*	OR	95%CI	*p*
**Country**					**0.002**			**<0.001**
Belgium	169	37.0	Ref			Ref		
Netherlands	808	50.2	1.34	1.07–1.68	**0.012**	1.44	1.14–1.82	**0.003**
Germany	322	38.7	1.01	0.79–1.29	0.94	1.09	0.84–1.41	0.51
**Change in cross-border visits per month between autumn 2021 and before the pandemic**					**<0.001**			**<0.001**
No change	548	38.3	Ref			Ref		
Decrease of >1	548	47.8	1.96	1.51–2.55	**<0.001**	1.84	1.41–2.41	**<0.001**
Increase of >1	203	62.7	0.68	0.55–0.83	**<0.001**	0.69	0.56–0.84	**<0.001**
**Number of pre-pandemic border crossings per month**					**<0.001**			**<0.001**
1–2	430	32.6	Ref			Ref		
3–5	418	47.4	2.07	1.70–2.52	**<0.001**	1.91	1.56–2.34	**<0.001**
>5	451	64.4	4.68	3.67–5.97	**<0.001**	3.91	3.02–5.08	**<0.001**
**Sex**					0.14			
Female	717	42.2						
Male	582	48.4	1.13	0.96–1.32	0.14			
**Age group**					**0.002**			
18–29	114	46.7	1.37	0.99–1.91	0.062			
30–39	136	45.9	1.38	1.01–1.89	**0.043**			
40–49	196	46.7	1.34	1.01–1.78	**0.044**			
50–59	336	47.3	1.25	0.98–1.61	0.078			
60–69	324	41.1	0.90	0.71–1.16	0.42			
≥70	193	43.7	Ref					
**Level of education**					**0.047**			
Theoretical	643	46.7	Ref					
Practical	656	43.1	0.86	0.73–1.00	**0.047**			
**Work situation**					**<0.001**			**0.005**
Working in own country	730	45.8	Ref			Ref		
Working in other country	51	75.0	2.09	1.16–3.77	**0.014**	2.21	1.21–4.01	**0.010**
Not working	518	41.9	0.79	0.67–0.93	**0.003**	0.88	0.74–1.03	0.11
**Presence of comorbidities**					0.92			
No comorbidities	524	45.2	Ref					
Comorbidities	775	44.5	0.99	0.85–1.16	0.92			
**Having family/friends across the border**					**0.001**			**0.002**
No	613	37.8	Ref			Ref		
Yes	686	53.7	1.31	1.11–1.55	**0.001**	1.31	1.10–1.55	**0.002**
**Perceived difficulty (R1)**								
**Limit group size**					**<0.001**			**<0.001**
(very) easy/neutral	978	41.5	Ref			Ref		
(very) difficult	321	58.9	1.97	1.62–2.41	**<0.001**	1.54	1.24–1.91	**<0.001**
**Minimalize travel**					**<0.001**			**<0.001**
(very) easy/neutral	1,027	41.4	Ref			Ref		
(very) difficult	272	65.2	2.60	2.07–3.27	**<0.001**	1.99	1.55–2.56	**<0.001**
**Work from home** ^**A**^					**0.009**			
(very) easy/neutral/ not applicable	914	43.4	Ref					
(very) difficult	385	48.5	1.26	1.06–1.49	**0.009**			
**Perceived usefulness (R1)**
**Limit group size**					**<0.001**			
(very) useful/neutral	1,134	43.4	Ref					
(very) useless	165	57.5	1.64	1.26–2.12	**<0.001**			
**Minimalize travel**					**<0.001**			**0.007**
(very) useful/neutral	1,168	43.3	Ref			Ref		
(very) useless	131	64.2	2.23	1.63–3.05	**<0.001**	1.58	1.13–2.21	**0.007**
**Work from home** ^**A**^					**0.011**			
(very) useful/neutral/not applicable	1,196	44.0	Ref					
(very) useless	103	56.9	1.52	1.10–2.10	**0.011**			

Reference = no negative experience of border restrictions. OR, odds ratio; 95%CI = 95% confidence interval. Nagelkerke pseudo-R^2^ value of the multivariable model = 0.17. Decrease > 1 indicates a decrease of 1 or more cross-border visits per month in autumn 2021, compared to before the COVID-19 pandemic. Increase > 1 indicates an increase of 1 or more cross-border visits per month in autumn 2021, compared to before the COVID-19 pandemic. R1, round 1 questionnaire. ^A^For ‘work from home’, all participants who were not working (e.g., retired, students) were grouped ‘not applicable’. This group was then included in the categories of ‘(very) useful or neutral’ for perceived usefulness, and ‘(very) easy or neutral’ for perceived efficacy of working from home.

The adjusted odds ratios for a negative experienced impact of border restrictions was 1.4 (95%CI 1.1–1.8) for Dutch participants, compared to Belgian. Those with a decrease had a 1.8 odds (95%CI 1.4–2.4) and those with an increase a 0.7 odds (95%CI 0.6–0.8) for a negative experienced impact. Odds for negative experience increased as pre-pandemic cross-border visits were higher (3–5 visits: aOR = 1.9; 95%CI 1.6–2.3 and ≥ 6 visits: aOR = 3.9; 95%CI = 3.0–5.1). Working across the border increased the likelihood of a negative experience (aOR = 2.2; 95%CI = 1.2–4.0). Odds for negative impact were 1.3 times higher (95%CI 1.1–1.6) when having family, friends or acquaintances across the border. Difficulty adhering to ‘minimalize travel’ resulted in twice the odds for a negative experience with border restrictions (aOR = 2.0; 95%CI 1.6–2.6), while a low perceived usefulness had an odds of 1.6 (95%CI 1.1–2.2). Difficulty adhering to ‘limit group size’ presented a 1.5 odds (95%CI 1.2–1.9) for a negative experience (Table 5).

### CBM and happiness

**Figure 4:**
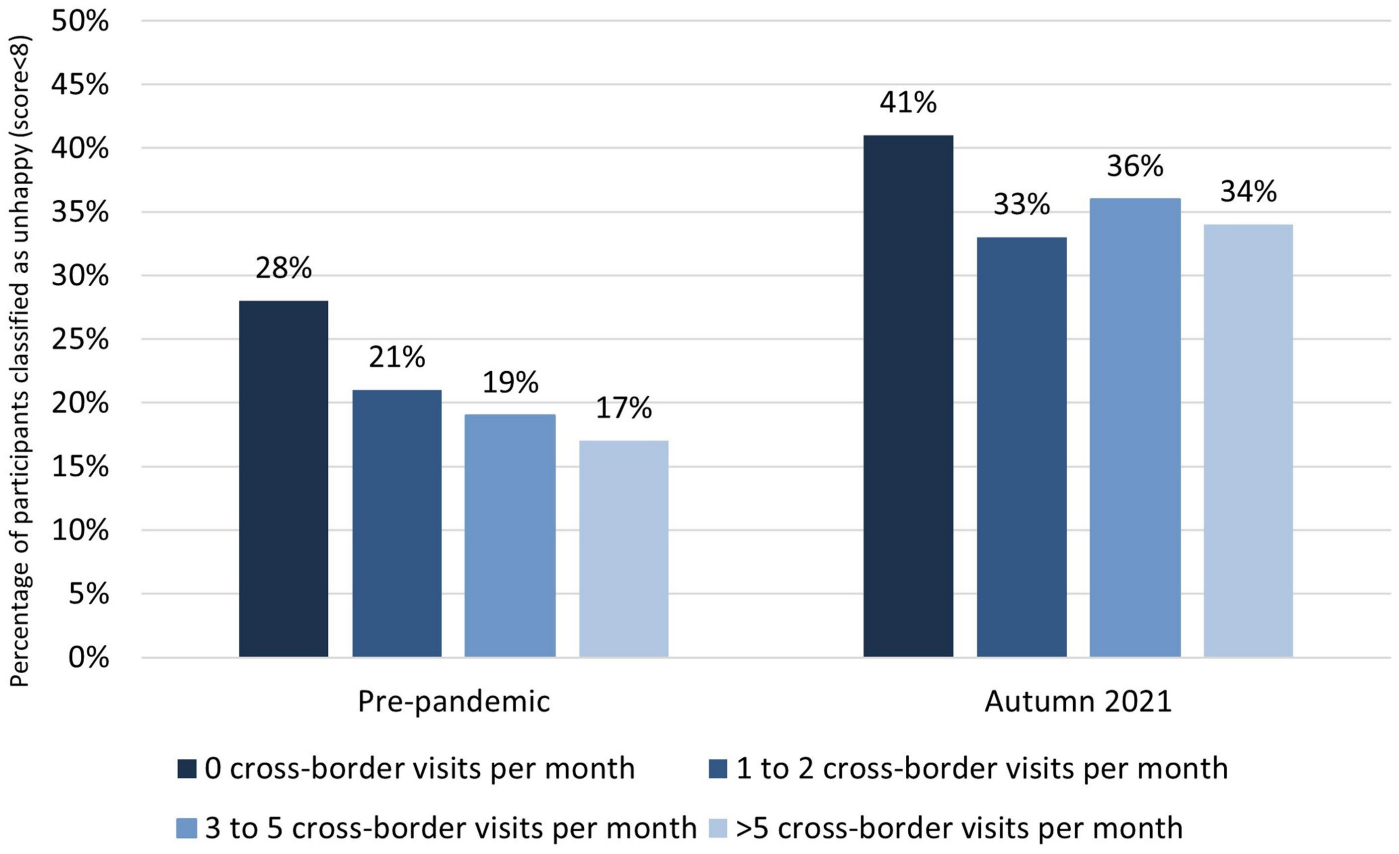
Proportion of participants classified as unhappy (happiness score < 8) pre-pandemic and in autumn 2021, compared for the number of cross-border visits per month (0 visits, 1–2 visits, 3–5 visits, and > 5 visits; *N* = 3,540). ***p < 0.001; **p < 0.01; *p < 0.05 for logistic regression analysis corrected for country, age, sex, and having social contacts across the border. Nagelkerke pseudo-*R^2^* value = 0.031.

Pre-pandemic, 28% of participants with no CBM were classified as unhappy, compared to 17% of participants with >5 cross-border visits per month. In autumn 2021, proportions of unhappiness ranged between 33 and 36% for participants with CBM, and was 41% for participants with no CBM (Figure 4).

Adjusted logistic regression analyses indicated statistically significant differences between the ‘0 visits’ group and the other groups pre-pandemic [1–2 visits: aOR = 0.68 (95%CI 0.54–0.85); *p* < 0.001; 3–5 visits: aOR = 0.55 (95%CI 0.43–0.72); *p* < 0.001; >5 visits: aOR = 0.45 (95%CI 0.34–0.61); *p* < 0.001].

In autumn 2021, a statistically significant difference was observed between the ‘0 visits’ group and the ‘1–2 visits’ and ‘>5 visits’ groups [1–2 visits: aOR = 0.74 (95%CI 0.61–0.90); *p* = 0.002; 3–5 visits: aOR = 0.90 (95%CI 0.74–1.11); *p* = 0.32; >5 visits: aOR = 0.83 (95%CI 0.69–1.00); *p* = 0.049; Figure 4].

## Discussion

### Summary

This study examined changes in CBM and the impact of border restrictions in the EMR during the COVID-19 pandemic. Pre-pandemic, CBM was frequent, reported by 82%, with the highest proportion reported among Dutch participants. In autumn 2021, 40% of participants with pre-pandemic CBM, had decreased their number of cross-border visits (35% reduced CBM for everyday activities and 26% for visiting social contacts), while 49% had no change or a minor change, and 11% had an increase. A negative experienced impact of border restrictions was reported by 45% of participants who had pre-pandemic CBM. Negative experience was associated with Dutch residency, working across the border, having social contacts across the border, and finding measures useless or difficult to adhere to. CBM was associated with being happy, both pre-pandemic and during the pandemic, in autumn 2021.

### Pre-pandemic CBM and changes in CBM

This study provides unique insights as it is the first to investigate changes in CBM for visiting social network members and everyday activities in the EMR. Pre-pandemic, CBM was high (82%) among participants, with some variation in the three countries, with the highest rate in Germany and especially in Dutch participants. The latter observation might be explained by the fact that Dutch participants lived closest to a border; and as a result also might have been more impacted by border restrictions. Overall, 31% had at least one visit per month to cross-border social network members and 79% had at least one visit for everyday activities per month. The assessment of pre-pandemic CBM in the current study allowed for improved understanding of changes in CBM during the pandemic.

A substantial proportion of participants decreased their number of cross-border visits during the pandemic. Prior research in 25 country pairs using phone and Facebook similarly demonstrated a sharp decline in CBM of up to 82% compared to pre-pandemic levels (28). Compared to Belgium and German participants, Dutch participants more frequently showed an increase in both overall visits and short visits, adjusted for other characteristics. A previous study showed that Dutch citizens were less likely to restrict CBM for everyday activities or social contacts, compared to Belgian citizens (29). This can pose challenges for implementing policies, as cultural differences impact people’s responses to measures in different places. It was suggested that culture can influence pandemic-related behaviors, even apart from individual beliefs (29). To demonstrate, we previously showed that in the EMR, compliance and evaluation of COVID-19 measures, as well as intention to take the COVID-19 booster vaccine, were highest among German citizens and lower among Dutch citizens (30, 31).

Expectedly, finding travel-related measures difficult to adhere to or useless was associated with the CBM outcomes in the present study. Previous research has also linked beliefs on policies’ effectiveness to a greater likelihood of engaging in the associated behaviors (29). Other factors such as (health) literacy and communication of measures are also associated with compliance (32).

Despite the absence of CBM restrictions in autumn 2021, CBM remained lower compared to the pre-pandemic period, suggesting the presence of longer-lasting effects and structural changes in citizens’ mobility behavior. These changes likely resulted from the necessary adaptations in routines during the pandemic when work, school, family situations, and social interactions were disrupted (33).

### Impact of border restrictions

Overall, a significant portion of participants (45%) reported a negative experienced impact of border restrictions, with the highest proportion among Dutch participants. Approaches to border restrictions varied per country, with Belgium physically closing its borders, Germany implementing border checks, and the Netherlands only discouraging CBM. CBM has been linked to wellbeing and happiness, in line with findings of the current study. Previous research has shown links between free movement and experienced health, as daily mobility and spatial behavior can influence individuals’ wellbeing and happiness (10, 33). Negative experiences with border restrictions have been associated with worse mental health outcomes in Australia (34). Additionally, a report on 20 case studies conducted in several European border regions indicated that border restrictions had a negative impact on residents’ personal lives as families living on both sides of the border were divided (15). EMR-residents also faced challenges in accessing up-to-date country-specific information and keeping track of constantly changing rules, highlighting a need for improved communication and access to information (16, 17).

Having social contacts (friends or family) across the border was a crucial independent factor associated with a negative experience with border restrictions. It has been argued that the social isolation resulting from the pandemic and its measures worsened existing public health challenges (35). People’s social network size and composition were impacted by the pandemic. For example, a study in the Dutch subregion showed that network size and the number of emotional and practical supporters decreased, with a shift toward a smaller more family-oriented network (23). National policies tend to overlook the interconnected social lives of residents in border regions, which extend beyond geographical borders (5). Therefore, policies that prevent loss of social contact, as well as sustainable cross-border communication and collaboration, are essential to ensure effective pandemic management in border regions.

### Strengths and limitations

A main strength of the current study is the standardized data collection across the three countries, ensuring consistency in methodology and timing, along with the accessibility of questionnaires in all three languages. A limitation is the absence of information on the actual distance of residence and a border. It is plausible that participants residing closer to the border experienced a greater impact from border restrictions, as is likely for the Dutch participants. Among the EMR regions included in this study, the Dutch subregion has the smallest surface area, with most municipalities bordering Belgium or Germany, possibly resulting in higher rates of CBM and experienced negative impact among Dutch participants.

Another limitation is that pre-pandemic CBM was assessed in spring 2021, which may have been a potential for recall bias.

A further limitation is that we did not obtain information on which countries were predominantly visited (for example Dutch participants reporting CBM may have visited either Germany or Belgium). Regions with existing cultural or language separation might have been less affected, for example the experienced impact may have been different for Liège (French-speaking) compared to Dutch-or German-speaking regions in Belgium. A final limitation is that, while we have shown associations, no causal effects can be determined.

### Implications

Fostering social contact, such as by maintaining mobility, is not usually included in public health policy or pandemic preparedness, giving room for improvement.

Furthermore, policies are generally shaped at the national level rather than the regional level. Regions may place a higher emphasis on cross-border collaboration, experiencing the repercussions of lacking such policies, while it might be perceived as less urgent at the national level. Currently, cross-border collaboration mainly occurs at the operational level. Prevention officers, united in a cross-border collaboration know each other and establish direct contact during crises like COVID-19. However, this interaction is largely driven by personal connections rather than being supported by a structured government policy. Further investigation is needed to explore the added value of these collaborative networks. The present initiative for cross-border cooperation is largely driven by practical needs. Whether this level should also lead the initiation of national policies, should be further examined.

To make informed decisions about implementing, adjusting, easing, or suspending border restrictions, it is crucial to assess not only their effectiveness but also any unintended consequences, such as adverse impact on social, mental, and physical health, that may arise (36).

As the virus enters an endemic phase, it is important to update legal frameworks and establish enforceable measures to prepare for possible public health threats in the future. It has been established that in a highly globalized world and a Europe with open borders, imposing border restrictions is detrimental and should only be implemented in crisis situations (37). Border restrictions should be temporary, proportionate, and coordinated between countries, rather than a unilateral action. Targeted measures are considered more effective and less disruptive (37).

It has been suggested that border-related movements are not inherently more risky for SARS-CoV-2 transmission compared to in-country movements, and public health professionals have questioned the emphasis on restricting borders while leaving in-country movements largely unrestricted (5). If border restrictions or other measures are implemented, they should be aligned with neighboring countries to prevent adverse effects and impact on citizens (13, 37, 38). Therefore, cross-border collaboration on interventions that limit transmission but also minimize impact on citizens, especially in border regions, should be encouraged.

## Conclusion

The COVID-19 pandemic has had significant implications for CBM in the EMR. We highlight the importance of considering the unique circumstances of border regions and the substantial impact of border restrictions on citizens’ lives. Cross-border social networks, cross-border commuting, and the perception of measures played an important role in the changes observed in CBM and the negative experienced impact of border restrictions. Overall, our study shows the implications of the pandemic on CBM and emphasizes the importance of collaborative policies. In this way, public health goals can be prioritized while mitigating disruptions to citizens’ lives and preserving the well-being of border regions.

## Data availability statement

The datasets presented in this article are not readily available because the data of this study contain potentially identifying and sensitive participant information. Due to the General Data Protection Regulation, it is not allowed to distribute or share any personal data that can be traced back (direct or indirect) to an individual. In addition, publicly sharing the data would not be in accordance with participants’ consent obtained for this study. Therefore, data used and/or analyzed during the study are available from the head of the data-archiving of the Public Health Service South Limburg on reasonable request. Interested researchers should contact the head of the data-archiving of the Public Health Service South Limburg (Tamara Kleine: tamara.kleine@ggdzl.nl) when they would like to re-use data. Requests to access the datasets should be directed to tamara.kleine@ggdzl.nl.

## Ethics statement

The studies involving human participants were reviewed and approved by the Medical Ethical Committee of the MUMC+ (2020-2463-A-1). In Belgium, the study was approved by the Medical Ethical Committee of the University Hospital Ghent and the University of Ghent (BC-09754). In Germany, no ethical assessment of the project was required. Written informed consent was obtained from the individual(s) for participation and for the publication of any potentially identifiable images or data included in this article.

## Author contributions

CB: Conceptualization, Formal analysis, Investigation, Methodology, Writing – original draft, Writing – review & editing. SB: Conceptualization, Methodology, Supervision, Writing – review & editing. CH: Conceptualization, Funding acquisition, Methodology, Supervision, Writing – review & editing. CS: Investigation, Writing – review & editing. CM: Investigation, Writing – review & editing. SD: Funding acquisition, Writing – review & editing. DH: Writing – review & editing. IL: Writing – review & editing. PS: Writing – review & editing. DP: Funding acquisition, Writing – review & editing. BZ: Funding acquisition, Writing – review & editing. ND-M: Conceptualization, Methodology, Supervision, Writing – review & editing.
