# Supplementary material for: Cross-border mobility in the Meuse-Rhine Euroregion: impact of COVID-19 border restrictions on everyday activities and visiting social network members
Source: Front Public Health. 2024 Apr 18;12:1281072. doi: 10.3389/fpubh.2024.1281072 (PMC11079879; doi:10.3389/fpubh.2024.1281072)
Supplement: Supplementary file 1 [file Table_1.DOCX]

# Supplementary materials

**Supplementary table 1.** Univariate and multivariable logistic regression analysis investigating factors associated with baseline CBM (before the pandemic; N=3543).

|  | **Univariate-corrected for country** | | | **Multivariable** | | |
| --- | --- | --- | --- | --- | --- | --- |
|  | **OR** | **95% CI** | **p** | **OR** | **95% CI** | **p** |
| **Country** |  |  | **<0.001** |  |  | **<0.001** |
| Belgium | Ref |  |  | Ref |  |  |
| Netherlands | 5.16 | 4.16-6.40 | **<0.001** | 4.35 | 3.47-5.45 | **<0.001** |
| Germany | 2.45 | 1.97-3.05 | **<0.001** | 2.42 | 1.94-3.04 | **<0.001** |
| **Sex** |  |  | 0.65 |  |  |  |
| Female | Ref |  |  |  |  |  |
| Male | 0.96 | 0.80-1.15 | 0.65 |  |  |  |
| **Age group** |  |  | **<0.001** |  |  |  |
| 18-29 vs ≥70 | 1.11 | 0.79-1.56 | 0.56 |  |  |  |
| 30-39 vs ≥70 | 1.56 | 1.10-2.20 | **0.012** |  |  |  |
| 40-49 vs ≥70 | 1.92 | 1.39-2.66 | **<0.001** |  |  |  |
| 59-59 vs ≥70 | 1.58 | 1.20-2.09 | **0.001** |  |  |  |
| 60-69 vs ≥70 | 1.52 | 1.16-2.00 | **0.003** |  |  |  |
| ≥70 | Ref |  |  |  |  |  |
| **Level of education** |  |  | **0.021** |  |  |  |
| Theoretical | Ref |  |  |  |  |  |
| Practical | 0.81 | 0.68-0.97 | **0.021** |  |  |  |
| **Work situation** |  |  | **<0.001** |  |  | **<0.001** |
| Working in own country | Ref |  |  | Ref |  |  |
| Working in other country | 6.62 | 2.04-21.43 | **0.002** | 2.74 | 0.84-9.01 | 0.096 |
| Not working | 0.65 | 0.55-0.78 | **<0.001** | 0.63 | 0.53-0.76 | **<0.001** |
| **Presence of comorbidities** |  |  | 0.53 |  |  |  |
| No comorbidities | Ref |  |  |  |  |  |
| Comorbidities | 0.94 | 0.79-1.13 | 0.53 |  |  |  |
| **Having family/friends/acquaintances across the border** |  |  | **<0.001** |  |  | **<0.001** |
| No | Ref |  |  | Ref |  |  |
| Yes | 4.57 | 3.57-5.86 | **<0.001** | 4.41 | 3.43-5.66 | **<0.001** |

OR for CBM at baseline vs. no CBM at baseline.

OR=odds ratio; 95%CI=95% confidence interval. Nagelkerke pseudo-*R^2^* value of the multivariable model=0.19.

**Supplementary table 2.** Proportions on CBM for participants with pre-pandemic CBM per EMR-country (N=2900).

|  | **Netherlands (N=1610)** | **%** | **Belgium (N=457)** | **%** | **Germany**  **(N=833)** | **%** |
| --- | --- | --- | --- | --- | --- | --- |
| **CBM pre-pandemic** |  |  |  |  |  |  |
| Yes, social visits and visits for everyday activities | 644 | 40.0 | 118 | 25.8 | 239 | 28.7 |
| Yes, only visits for everyday activities | 909 | 56.5 | 329 | 72.0 | 568 | 68.2 |
| Yes, only social visits | 57 | 3.5 | 10 | 2.2 | 26 | 3.1 |
| **Number of pre-pandemic border crossings per month** |  |  |  |  |  |  |
| 1-2 | 593 | 36.8 | 286 | 62.6 | 439 | 52.7 |
| 3-5 | 543 | 33.7 | 94 | 20.6 | 245 | 29.4 |
| ≥6 | 474 | 29.4 | 77 | 16.8 | 149 | 17.9 |
| **CBM in round 1**  **(spring 2021)** |  |  |  |  |  |  |
| Yes, social visits and visits for everyday activities | 126 | 7.8 | 43 | 9.4 | 49 | 5.9 |
| Yes, only visits for everyday activities | 422 | 26.2 | 97 | 21.2 | 198 | 23.8 |
| Yes, only social visits | 85 | 5.3 | 13 | 2.8 | 32 | 3.8 |
| No | 977 | 60.7 | 304 | 66.5 | 554 | 66.5 |
| **CBM in round 2**  **(autumn 2021)** |  |  |  |  |  |  |
| Yes, social visits and visits for everyday activities | 338 | 21.0 | 65 | 14.2 | 122 | 14.6 |
| Yes, only visits for everyday activities | 910 | 56.5 | 199 | 43.5 | 413 | 49.6 |
| Yes, only social visits | 71 | 4.4 | 16 | 3.5 | 21 | 2.5 |
| No | 291 | 18.1 | 177 | 38.7 | 277 | 33.3 |
| **Having family/friends across border** |  |  |  |  |  |  |
| In the Netherlands | - | - | 127 | 27.8 | 216 | 25.9 |
| In Belgium | 566 | 35.2 | - | - | 152 | 18.2 |
| In Germany | 455 | 28.3 | 52 | 11.4 | - | - |
| No | 788 | 48.9 | 298 | 65.2 | 537 | 64.5 |
| **Work in other country** |  |  |  |  |  |  |
| Total | 26 | 1.6 | 31 | 6.8 | 12 | 1.4 |
| In the Netherlands | - | - | 23 | 5.0 | 11 | 1.3 |
| In Belgium | 5 | 0.31 | - | - | 1 | 0.12 |
| In Germany | 15 | 0.93 | 7 | 1.5 | - | - |
| Other | 6 | 0.37 | 1 | 0.22 | 0 | 0 |
